# Dynamics of inflammatory cytokine expression in bovine endometrial cells exposed to cow blood plasma small extracellular vesicles (sEV) may reflect high fertility

**DOI:** 10.1038/s41598-023-32045-1

**Published:** 2023-04-03

**Authors:** Pevindu Abeysinghe, Natalie Turner, Eman Mosaad, Jayden Logan, Murray D. Mitchell

**Affiliations:** School of Biomedical Sciences, Faculty of Health, Centre for Children’s Health Research, Queensland University of Technology, Brisbane, QLD 4029 Australia

**Keywords:** Diagnostic markers, Prognostic markers, Cell biology, Genetics, Immunology, Molecular biology, Diseases, Medical research

## Abstract

Aberrant inflammation in the endometrium impairs reproduction and leads to poor fertility. Small extracellular vesicles (sEV) are nanoparticles 30–200 nm in-size and contain transferable bioactive molecules that reflect the parent cell. Holstein–Friesian dairy cows with divergent genetic merit, high- (n = 10) and low-fertile (n = 10), were identified based on fertility breeding value (FBV), cow ovulation synchronization and postpartum anovulatory intervals (PPAI). In this study, we evaluated the effects of sEVs enriched from plasma of high-fertile (HF-EXO) and low-fertile (LF-EXO) dairy cows on inflammatory mediator expression by bovine endometrial epithelial (bEEL) and stromal (bCSC) cells. Exposure to HF-EXO in bCSC and bEEL cells yielded lower expression of PTGS1 and PTGS2 compared to the control. In bCSC cells exposed to HF-EXO, pro-inflammatory cytokine IL1-α was downregulated compared to the untreated control, IL-12α and IL-8 were downregulated compared to the LF-EXO treatment. Our findings demonstrate that sEVs interact with both endometrial epithelial and stromal cells to initiate differential gene expression, specifically genes relate to inflammation. Therefore, even subtle changes on the inflammatory gene cascade in the endometrium via sEV may affect reproductive performance and/or outcomes. Further, sEV from high-fertile animals acts in a unique direction to deactivate prostaglandin synthases in both bCSC and bEEL cells and deactivate pro-inflammatory cytokines in the endometrial stroma. The results suggest that circulating sEV may serve as a potential biomarker of fertility.

## Introduction

The maternal reproductive system interacts with the immune system by maintaining a unique equilibrium during pregnancy and different stages of reproductive cycle^1^. Expression levels of pro-inflammatory and anti-inflammatory cytokines in the endometrium controls vital reproduction functions such as ovulation, implantation, and parturition^2^. During first stages of pregnancy, expression levels of pro-inflammatory cytokines such as interleukin-1-alpha (IL-1α), Interleukin-8/C-X-C motif ligand 8 (IL-8/CXCL8) upregulate to facilitate implantation and placentation, but subsequently, an anti-inflammatory state switches on to assist growth of the foetus^3^. Upregulation of anti-inflammatory Interleukin-4 (IL-4), interleukin-10 (IL-10) and C-X3-C motif chemokine ligand 1 (CX3CL1) cytokine expression ensures symbiotic relationship between the foetus, placenta, and the maternal reproductive system^3–5^. Finally, an activation of pro-inflammatory state prior to the labour facilitates successful foetal delivery^6,7^. During this stage, cyclooxygenases, prostaglandin-endoperoxide synthase-1 (PTGS1)/cyclooxygenase-1 (COX-1) and prostaglandin-endoperoxide synthase-2 (PTGS2)/ cyclooxygenase-2 (COX-2) expression levels upregulate in the endometrium as well. Thus, dynamic balance of inflammatory cytokines in the endometrium is influential during pregnancy^8^, fertility state^9^ and birth outcomes^10^.

Small extracellular vesicles (sEV) are membrane bound heterogeneous group of 30–200 nm sized nanovesicles and exosomes (30–150 nm) are a niche subgroup of sEV which facilitate intercellular communication via unique bioactive molecules such as proteins^11^, miRNAs^12^, and eicosanoids^13^. Relationships of exosome derived miRNA^14,15^, protein^16^ and eicosanoids^17^ specifically on fertility have been revealed recently, identifying the severity of the genetic behavior of exosomal cargo. Biogenesis of sEV and associated cargo loading mechanisms facilitate formation of sEV unique to their cellular origin and provides significant information on the cellular microenvironment^18,19^. Circulating sEV release into systemic blood circulation system^20^ to interact with the recipient cells by activating membrane receptors and releasing their cargo into recipient cells^21^. For example, exosomes contained in blood plasma interact with cells in the local and distant environment as they travel within systemic circulation, and once in contact with a recipient cell are taken up by active endocytosis or activation of membrane receptors^22^. For example, a highly vascular organ such as the bovine uterus could indeed be under the effect of circulating exosomes via direct blood supply. Uptake of exosomal molecular cargo may occur on the basal side of cells lining the endometrial cavity, bovine endometrial epithelial (bEEL) and stromal (bCSC) cells, and may result in alterations to inflammation-related genes, as has been modelled previously in vitro^17^. Information encrypted in sEV cargo potentially modify the phenotype and the molecular functionality of the recipient cells, such as immune function. sEV alters innate and adaptive immune responses specifically in cellular microenvironment acting as decoys of immune checkpoint inhibition. Therefore, multifaceted role of sEV and exosome research have emerged as study areas to understand vital cellular and molecular processes such as immune function, which further expands into biomarker development and targeted therapeutics^23^.

Compromised uterine immune system leads to severe reproduction disorders which later challenges the fertility. Endometriosis affects reduces the reproduction capacity of around 10% of women in their reproductive age globally which defines as the growth of endometrium outside the uterus due to aberrant inflammation^24^. Inflammatory chronic endometritis is a major contributor of recurrent implantation failure (RIF) and recurrent pregnancy loss by changing the optimal endometrial immune environment^25,26^. An activated inflammatory system triggers reproductive infectious disorders in ruminants such as dairy cows^27^, in which affected low fertile animals being an economic and welfare burden to animal management systems^28^. Metritis and mastitis are common inflammatory diseases increase metabolic pressure in the reproductive tract of dairy cows and eventually leads to infertility^29^. Circulating sEV transports immune related signals through the systemic circulation, thus, a critical relationship between sEV and immune related functions such as inflammation exists. As such, during pregnancy sEV modulates maternal immunological responses including metabolic adaptations^30^. Nonetheless, exosomes in blood circulation increase across gestation to facilitate maternal–fetal crosstalk through the placenta^31,32^.

Circulating plasma sEV and exosomes has been identified as biomarkers of transition cows with metabolic dysfunction showing differential expression of PTGS1 and PTGS2 in bovine endometrial stromal (bCSC) and epithelial (bEEL) cells after incubating with circulating exosomes^17^. However, a retrospective study on how sEV affects the fertility states of animals has not been conducted so far. In the present study, we investigated the expression of pro-inflammatory and anti-inflammatory cytokine production in bCSC and bEEL endometrial cell lines incubated with circulating plasma sEV from high and low fertile dairy cow. This includes a comprehensive study on expression of prostaglandin catalysing agents, cytokines, and eicosanoids in bCSC and bEEL cells using custom-made qPCR arrays after incubation with sEV from dairy cows with divergent genetic merit for fertility.

## Materials and methods

### Animals

The animals, management, and sample collections were approved by the Ruakura Animal Ethics Committee (AEC 13574, 13934 and 14200) (AgResearch, Hamilton, New Zealand)^33^ and Holstein–Friesian dairy cows used in this study were part of a larger experiment of an established fertility animal model^33,34^. The study was conducted in compliance with ARRIVE guidelines and all methods were performed in accordance with the relevant guidelines and regulations^35^.

From the larger group of dairy cows, 52 cows were identified in 6 different groups based on fertility breeding value (BV), cow ovulation synchronization and postpartum anovulatory intervals (PPAI) which is the number of days from a cow calving to returning to oestrus (Table 1).

**Table 1:**
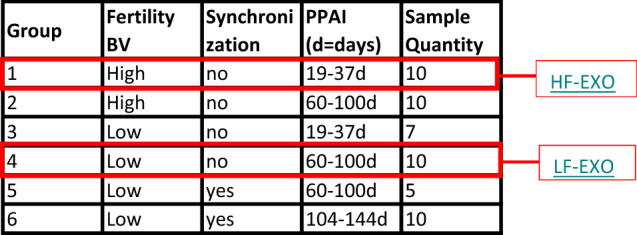
Classification of 52 dairy cows into 6 different groups by fe﻿rtility breeding value (BV), ovulation synchronization and postpartum anovulatory intervals (PPAI).

From these 6 groups, 20 cows were identified as being at two extreme diversities either high fertile, without synchronization and lowest PPAI—Group 1 (HF) (n = 10) and low fertile, without synchronization and highest PPAI—Group 4 (LF) (n = 10). These animals were kept similar in genetic characters for other key traits (e.g. body weight, milk production, and percentage of North American genetics) except from the fertility breeding values (FBV). Blood samples were collected into EDTA Vacutainer tubes (Greiner Bio-one, Kremsmunster, Austria) from a jugular vein straightaway or following a 48 h oestrus synchronization with prostaglandins. The blood samples were placed immediately on ice and centrifuged at 1500 × *rcf* for 12 min at 4 °C, and the plasma aspirated and stored at − 80 °C.

### Extracellular vesicle isolation from plasma by ultracentrifugation

EVs were isolated from a total of 20 blood plasma samples using as established sequential centrifugation protocol as previously described^36,37^. Briefly, first the plasma was centrifuged at 2000 × *rcf* for 30 min at 4 °C and 12,000 × *rcf* for 30 min at 4 °C to remove cellular debris and apoptotic bodies. Then the supernatant was filtered through a 0.22-μm filters (Corning Inc., Corning, NY) and ultracentrifuged at 100,000 × *rcf* for 2 h at 4 °C. Finally, the pellets containing the extracellular vesicles were resuspended in 500 μL of filtered Dulbecco’s Phosphate Buffered Saline (DPBS, pH 7.0–7.2; Gibco, Life Technologies Australia Pty Ltd) and stored at − 80 °C for further analysis.

### sEV enrichment by size-exclusion chromatography columns (SEC)

Extracellular vesicles obtained from ultracentrifugation were fractionated using qEV original size exclusion columns (Izon Science, New Zealand). Individual 500 µL fractions were eluted from the column and collected in separate 1.5 mL microcentrifuge tubes (a total of 16 fractions), as per manufacturer’s instructions. Then the sEV characterization experiments were conducted for individual SEC fractions to identify sEV/exosome markers. The fractions were collected as follow s; 1–6 as void volume and particles > 200 nm, 7–10 as exosomal (EX) fractions (particles < 200 nm), and 11–16 as soluble proteins (non-EX) fractions. Separate columns were used per animal groups to maintain group heterogeneity. In between uses, the columns were flushed with 0.5 mL 1 M NaOH solution, followed by 15–20 mL filtered DPBS.

### Protein quantification

Quantification of protein concentration of SEC fractions was performed using Bicinchoninic Acid (BCA) assay (Sigma-Aldrich, St Louis, MO, USA) and bovine serum albumin (Sigma-Aldrich, St Louis, MO, USA) dilutions were used as standards.

### Western blot

Individual fractions resulting from SEC were analysed by western blot for the presence of EV markers and contaminating plasma proteins. Visualisation of the residual bovine serum albumin (BSA) and sEV/exosome proteins were conducted for the collected SEC fractions F7–F16. Equal volumes (10 µL) of sample from the individual SEC fractions F7–F16 were aliquoted for WB analysis, as previously described^38^. Then the sEV/exosome protein enriched SEC fractions were selected as F7-F10 (EXO) and non-sEV/exosome protein (BSA) enriched SEC fractions as F11-F16 (non-EXO). Samples were dried in a vacuum concentrator (cat number 5305000380, Eppendorf Concentrator plus, Sydney, Australia) and resuspended to a final volume of 10.5 μl Briefly, 4 × NuPAGE LDS sample buffer (NP0007, Thermofisher Scientific, Brisbane, Australia) and 10 × NuPAGE sample reducing agent (NP0004, Thermofisher Scientific, Brisbane, Australia) were added to give a final concentration of 1 ×, and reduced for 10 min at 70 °C, as per the manufacturer’s instructions. For visualisation of Flotillin-1 (FLOT-1), CD81 in pooled sEV-enriched^7–10^ and non-sEV-enriched^11–16^ fractions, the same procedure was followed as for BSA, as previously described^36^. Samples were resolved by electrophoresis on NuPAGE™ 4 to 12%, Bis–Tris, 1.0 mm, Mini Protein Gels, 15-well (NP0336BOX, Thermofisher Scientific, Brisbane, Australia) with Chameleon^®^ Duo Pre-stained Protein Ladder (928–60000, Li-COR, Mulgrave, Australia). The protein gel was transferred onto a polyvinylidene fluoride membrane (Bio-Rad Laboratories Pty Ltd., Sydney, Australia) using the Trans-Blot Turbo system. Membranes were briefly washed in phosphate buffered saline containing 0.1% Tween-20 (PBST) (Sigma-Aldrich (Merck), Melbourne, Australia), before blocking in 5 mL Odyssey Intercept blocking buffer (927–70001, Li-COR, Mulgrave, Australia) and 5 mL phosphate buffered saline (PBS) (Sigma-Aldrich (Merck), Melbourne, Australia) for 1 h at RT. The primary antibody was diluted with 1:1 Odyssey Blocking buffer, PBS, and Tween-20 added to final concentration of 0.1%. Samples were incubated with primary antibody overnight; anti-BSA (1:5000 dilution, Rabbit polyclonal (ab192603, Abcam, Melbourne, Australia); recombinant anti-Flotillin-1 (1:1000 dilution, Rabbit monoclonal (ab133497, Abcam, Melbourne, Australia): anti-CD81 (1:500 dilution, Rabbit polyclonal (NBP1-77039, Novus Biologicals, LLC). The next day, membranes were washed four times in PBST for 5 min each, and the membranes were incubated with secondary antibody for 1 h at RT in the dark with gentle rocking: Goat anti-Rabbit IgG (1:15,000 dilution, Li-COR, Mulgrave, Australia). The secondary antibody was diluted with 1:1 Odyssey Intercept blocking buffer, PBS, and Tween-20 added to a final concentration of 0.1%. The membranes were washed in PBST four times for 5 min each. Membranes were rinsed briefly in PBS and imaged with Li-COR Odyssey fluorescent scanner at 700 and 800 nm. All images were processed using Image Studio Lite v5.2 (Li-COR Biosciences, Lincoln, NE, USA). Contrast and brightness were adjusted equally across entire images to best visualise protein bands.

### Transmission electron microscopy

Visualization of sEV particles from SEC fractions were conducted by JEOL 1400 transmission electron microscopy (JEOL, Sydney, Australia). sEV samples (5 µL) were added onto glow discharged copper grids (200 mesh) for 3 min. Next the grid was negatively stained with 1% uranyl acetate for 2 min, then briefly blotted with blotting paper to remove excess liquid. The samples were than visualized in JEOL 1400 transmission electron microscope operated at 100 kV, and images captured using a 2 K TVIPS CCD camera (TVIPS, Gauting, Germany).

### Nanoparticle tracking analysis

Based on the presence of exosomal markers, exosomal fractions 7–10 were pooled. Measurements of particle size and concentration were performed using a NanoSight NS500 instrument (NanoSight NTA 3.1 Build 3.1.46, Malvern Panalytical, Sydney, Australia) as previously described^18^. Synthetic (latex) beads of size 100 nm were used to perform instrument calibration at a 1:250 dilution in deionized water as previously described^36^.

### Bovine endometrial epithelial and stromal cell culture

Bovine endometrial epithelial (bEEL) and stromal (bCSC) cell lines^39,40^ were a kind gift from Professor Michel A. Fortier (Université Laval, Québec). The cells were grown in RPMI media (Gibco, Thermo Fisher Scientific Australia Pty Ltd, Scoresby Vic) containing sEV/exosome depleted 10% fetal bovine serum (Bovorgen, Interpath Services Pty Ltd, Australia), and incubated at 37 °C and 5% CO_2_. Experiments were conducted in media without fetal bovine serum^17^.

### Functional studies of sEV on endometrial cells

For the initial cell culture, bCSC cells were grown in a seeding density of 8000 cells per well and bEEL cells were grown in a seeding density of 35,000 cells per well. Then cells were incubated for 24 h grown in RPMI media (Gibco, Thermo Fisher Scientific Australia Pty Ltd, Scoresby Vic) containing 10% fetal bovine serum (Bovorgen, Interpath Services Pty Ltd, Australia). For the co-incubation experiment (treatment with sEV), FBS free RPMI media was used. Cells were incubated with RPMI media with no addition of sEV (No EXO control, for baseline measurements), or treated with HF-EXO (n = 10) or LF-EXO (n = 10) with 1 × 10^8^ particles per well for 24 h to analyze gene expression. We performed 3 well technical replicates per individual cow (n = 20) and two of 3 well technical replicates for untreated NO EXO control. sEV concentration for co-incubation^41^ and incubation time^42^ were chosen in reference to literature. Cell culture experiments were performed in triplicate per cell line. Cells and cultured media were collected and stored at − 80 °C until required for further analyses.

### RNA extraction and cDNA synthesis

First, all three technical replicates were pooled together of each individual cow sample and two groups of untreated control from the cell culture experiments. Finally, duplicates of each pooled sample were used to perform RNA isolations. Therefore, the final number of technical replicates used for RNA isolation, subsequent qRT-PCR experiments and statistical analysis were as follows: untreated NO EXO control (n = 4), HF-EXO (n = 20) and, LF-EXO (n = 20). RNA from the samples (bEEL and bCSC cells) were extracted accordingly to manufacturer’s protocol using a Rneasy Mini kit (Qiagen, Victoria, Australia). The concentration and purity of RNA was determined using a Nanodrop NanoDrop™ One Microvolume UV–Vis Spectrophotometer (Thermo Fisher Scientific, Wilmington, Delaware). 500 ng of RNA was reverse transcribed into complementary DNA (cDNA) using RT^2^ First Strand Kit (Cat no. 330404; Qiagen, Victoria, Australia).

### Eicosanoid enzymes and inflammatory mediator gene expression analysis

Gene expression was analyzed after 24 h of HF-EXO and LF-EXO treatments on bEEL and bCSC. Real-time PCR (RT-PCR) quantification of eicosanoid enzymes and inflammatory mediator gene expression was performed using a customized bovine RT^2^ Profiler PCR Array (Cat. no. 330171 CLAB39919; Qiagen, Victoria, Australia). The complete plate plan of the array is in Supplementary File 1. The reaction mixture was prepared using the RT^2^ SYBR Green ROX qPCR Mastermix (Cat. no. 330523; Qiagen, Victoria, Australia) following the manufacturer’s instructions. Briefly, RT-PCR was performed using the Applied Biosystems^®^ ViiA™ 7 Real-Time PCR System (Applied Biosystems^®^, Carlsbad, California). with an initial 2 min incubation at 50 °C and 10 min incubation at 95 °C followed by 40 cycles at 95 °C for 15 s and 60 °C for 60 s. The specificity of the RT-PCR products was confirmed by analysis of melting curves. Gene expression data which met the cycle threshold cut-offs (< 35) were analyzed. The endogenous control genes included on the array were Actin, beta (ACTB), TATA box binding protein (TBP), hypoxanthine phosphoribosyl transferase 1 (HPRT1) and glyceraldehyde 3-phosphate dehydrogenase (GAPDH). PCR reproducibility, reverse transcription efficiency and the presence of genomic DNA contamination were verified before analyzing further. Housekeeper genes were not changed with treatment. Gene expression results were normalized to the endogenous control genes ACTB, TBP, HPRT1 and GAPDH. Real-time PCR data were analyzed using comparative C_T_ method^17,43,44^.

### Gene enrichment analysis

PANTHER v17.0^35^ was used to perform gene ontology (GO) enrichment analysis for the target genes of DE genes and ggplot2 v3.4.0 (https://ggplot2.tidyverse.org/) of R package 4.2.2^45^. The search tool for retrieval of interacting genes (STRING) (https://string-db.org) database was utilized for generation of protein–protein interaction (PPI) network for the intersected target genes^46^. Using the STRING database, species “Bos taurus” were chosen to build a network model visualized by Cytoscape (v3.9.0)^47^. Protein subcellular localization was visualized using compartments feature in StringDB^48^.

### Statistical analysis

Data were imported and statistical analysis were performed in GraphPad Prism (version 9, GraphPad Inc., La Jolla, CA). Mann–Whitney test) was performed between for the BCA protein concentration and NTA total yield comparisons to identify significant difference between HF-Exo and LF-EXO groups. Values are presented as mean ± SD (**P < 0.001).

Data analysis for qRT-PCR gene expression results were performed first by identifying outliers using ROUT method with Q value of 5% for the cleaned data, Brown-Frosythe and Welch ANOVA test along with Dunnett’s T3 multiple comparison as post-hoc test was performed to identify statistical significance differences between the control, HF-EXO, and LF-EXO groups. Data are presented as sample means ± SEM. P-value of *P ≤ 0.05, **P ≤ 0.01, ***P ≤ 0.001 were considered statistically significant.

## Results

### LF-EXO shows higher total protein concentrations, however, did not show significant difference in total particle yield

**Figure 1:**
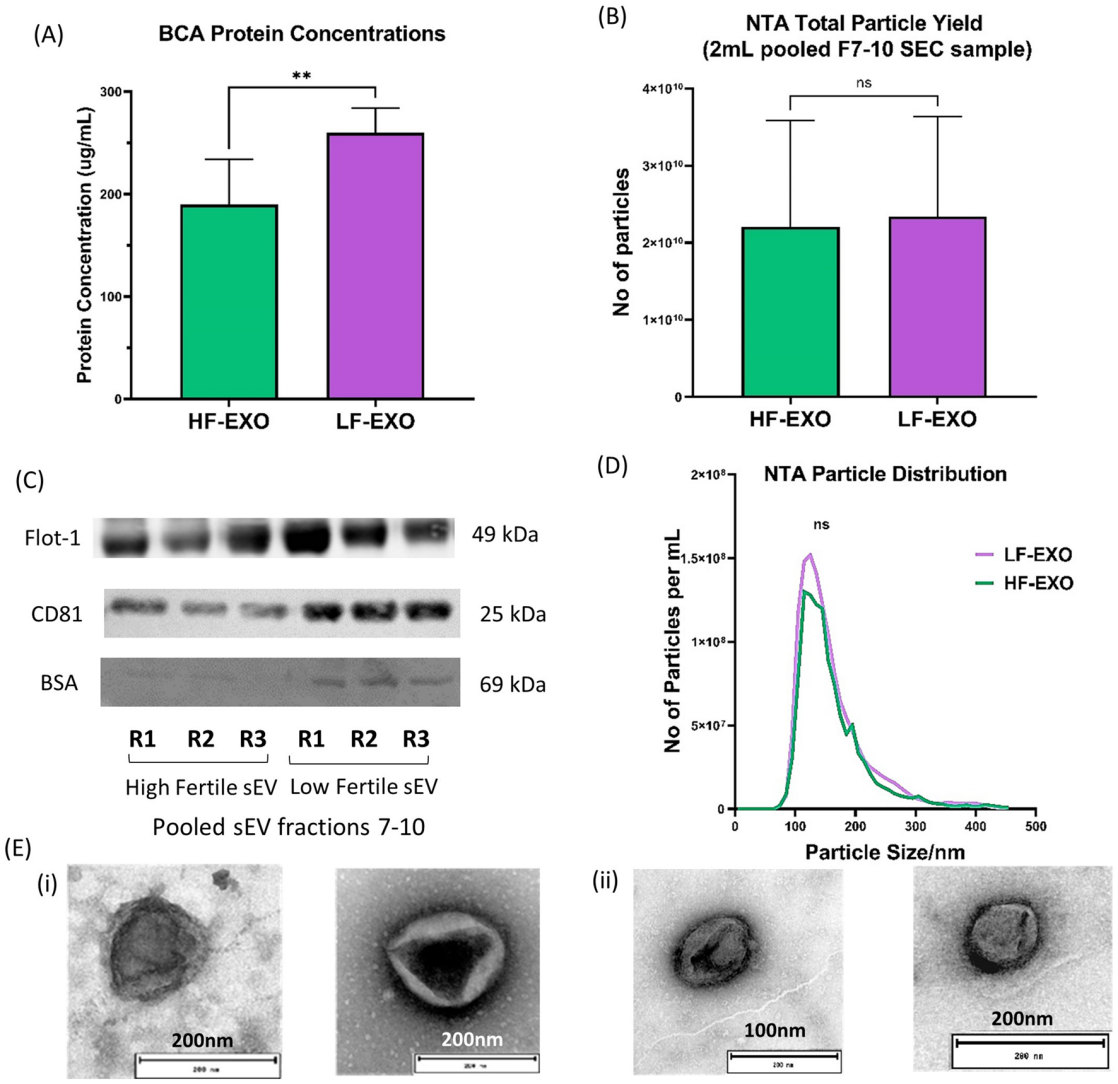
Blood plasma sEV from high fertile and low-fertile both confirmed sEV characteristics and differ in total protein concentrations. (**A**) Total protein content of sEV from low fertile dairy cow plasma are significantly higher than the high fertile. Values are presented as mean ± SD (**P < 0.001; Mann–Whitney test). (**B**) However, there’s no significant difference between the particle number (yield) according to the nano-particle tracking analysis (NTA) results. Values are presented as mean ± SD (**P < 0.001; Mann–Whitney test). (**C**) Representation of western blot sEV/exosome marker CD81, FLOT-1 and negative sEV/exosome marker BSA for pooled sEV fractions 7–10 of HF-EXO and LF-EX. Presence of FLOT-1 and CD81 and less abundance of BSA confirmed sEV/exosome. The full western blot images are available in the Supplementary File 3 (**D**) The size of sEV (nm) is within the defined size (30–200 nm)—average nano-particle tracking analysis (NTA) particle size distribution of HF-EXO and LF-Exo biological replicates (n = 10 each). There was no statistically significant difference between HF-EXO and LF-EXO particle size distributions (ns represents P > 0.05; Mann–Whitney test). (**E**) Spherical shape was confirmed in electron micrographs of exosomes from both (i) HF-EXO and (ii) LF-EXO.

The total protein content in sEV sample from LF-EXO was significantly higher than the HF-EXO as shown in the Fig. 1A. Interestingly, there was no significant difference in the total particle yield in LF-EXO in contrast to HF-EXO (Fig. 1B). Exosome protein marker CD81 signals were higher in LF-EXO compared to HF-EXO, however, non-exosome protein marker BSA was prominent in LF-EXO. BSA is the most abundant protein in cow blood plasma^49^, hence considered as a non-exosome protein marker to asses plasma protein contamination in sEV samples (Fig. 1C)^50^. Further sEV characterizations confirm the isolated sEV samples contain spherical cup-shaped particles with size ranges from 30 to 200 nm (Fig. 1D and E) which accepts the definition of exosomes. Nevertheless, presence of exosome protein markers Flot-1, CD81 in contrast to low abundance of BSA in both HF-EXO and LF-EXO ensures the characteristics of sEV.

### Incubation of blood plasma HF-EXO and LF-EXO on bovine endometrial stromal (bCSC) cells leads to changes in inflammatory related genes

**Figure 2:**
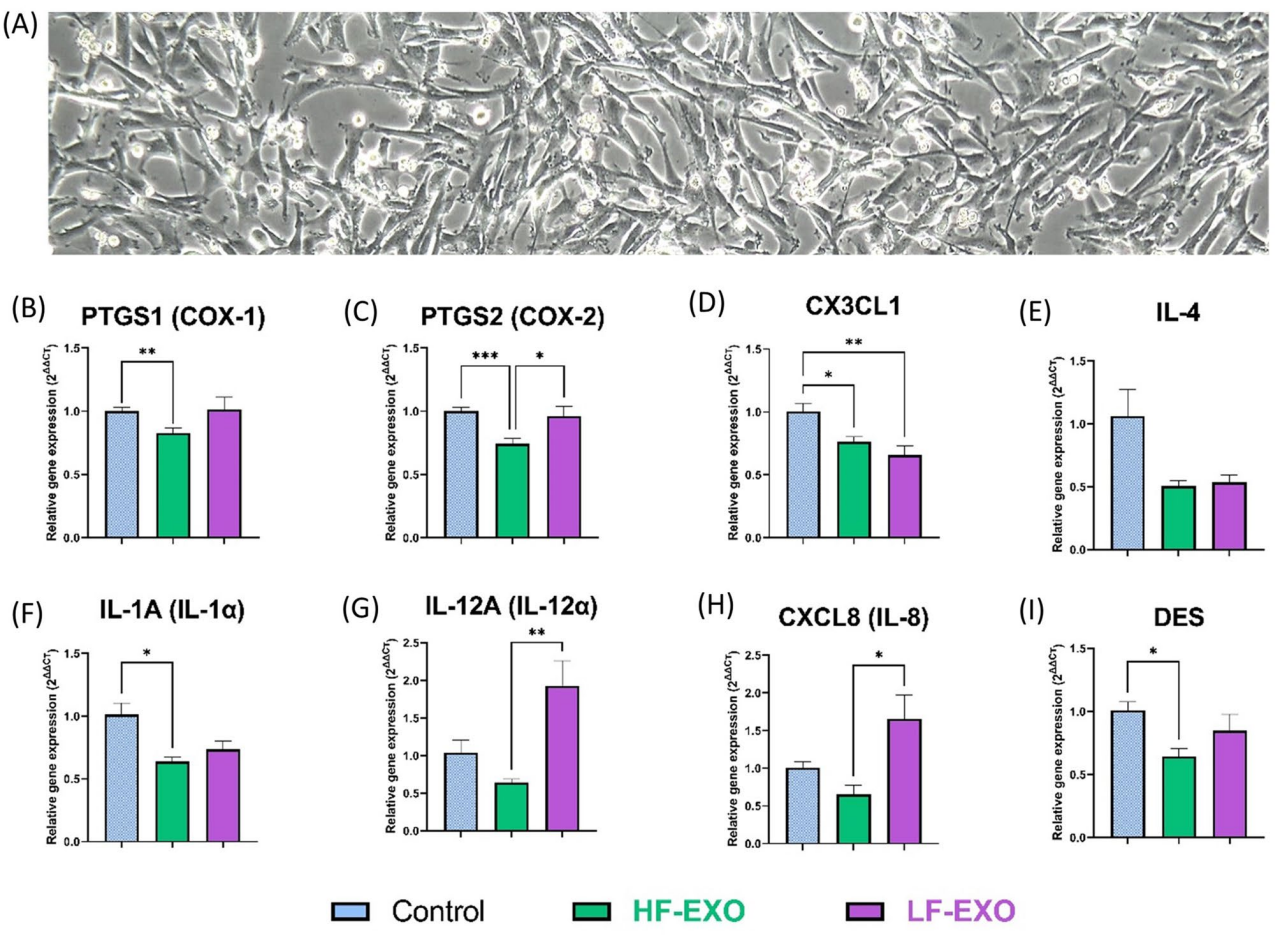
Co-incubation with sEV from dairy cows identified as high fertile (HF-EXO) and low fertile (LF-EXO) lead to differential gene expression in bovine endometrial stromal (bCSC) cells. (**A**) bCSC cells depicts fibroblast-like morphology and grow in multiple layers after confluency (at 48-h, 10X). (**B,C**) Prostaglandin synthase enzyme gene expression, (**D,E**) anti-inflammatory mediator gene expression (**F–I**) pro-inflammatory mediator gene expression in bCSC cells. GAPDH, ACTB and TBP were selected as the house-keeping genes to normalize the qRT-PCR data. Values are presented as mean ± SEM. Brown-Frosythe and Welch ANOVA test along with Dunnett’s T3 multiple comparison was selected as post-hoc test to identify statistical significance differences between the groups. *P ≤ 0.05, **P ≤ 0.01, ***P ≤ 0.001.

To investigate the effect of blood plasma sEV on the endometrial tissue, bovine endometrial stromal (bCSC) cells (Fig. 2A) were exposed to HF-EXO and LF-EXO, and then, inflammatory mediator gene expressions were assessed using real-time quantitative PCR (Fig. 2B–I).

Expression of pro-inflammatory cytokines in bovine stromal cells exposed to HF-EXO appeared to be downregulated when compared to the untreated control bovine endometrial stromal cells. Specifically, PTGS1, PTGS2, IL-1α and DES gene expressions were significantly low in HF-EXO treated bCSC in contrast to the control (Fig. 2B,C,F,I). Interestingly, PTGS2, which is one of the cyclooxygenases involve in inflammatory prostaglandins synthesis, was upregulated profoundly in bCSC cells exposed to LF-EXO while HF-EXO downregulated PTGS2 expression significantly (Fig. 2C). A similar trend was observed in IL-12α and IL-8 gene expressions between LF-EXO and HF-EXO treated bCSC cells (Fig. 2G–H).

However, anti-inflammatory cytokines did not show any significant differential expressions in bCSC cells treated with either LF-EXO or HF-EXO. Interestingly, when compared to the untreated control cells, CX3CL-1 (Fig. 2D) and IL-4 (Fig. 2E) anti-inflammatory gene expressions appeared to be downregulated by sEV incubation regardless of fertility status (a significant down-regulation was observed in CX3CL1 gene expression).

Therefore, more profound downregulation of pro-inflammatory gene expressions was observed in sEV with high fertility origin compared to the control in bovine endometrial stromal cells, and only a few significant changes were observed between HF-EXO and LF-EXO treated bCSC cells.

### Incubation of blood plasma HF-EXO and LF-EXO on bovine endometrial epithelial (bEEL) cells leads to changes in inflammatory related genes

**Figure 3:**
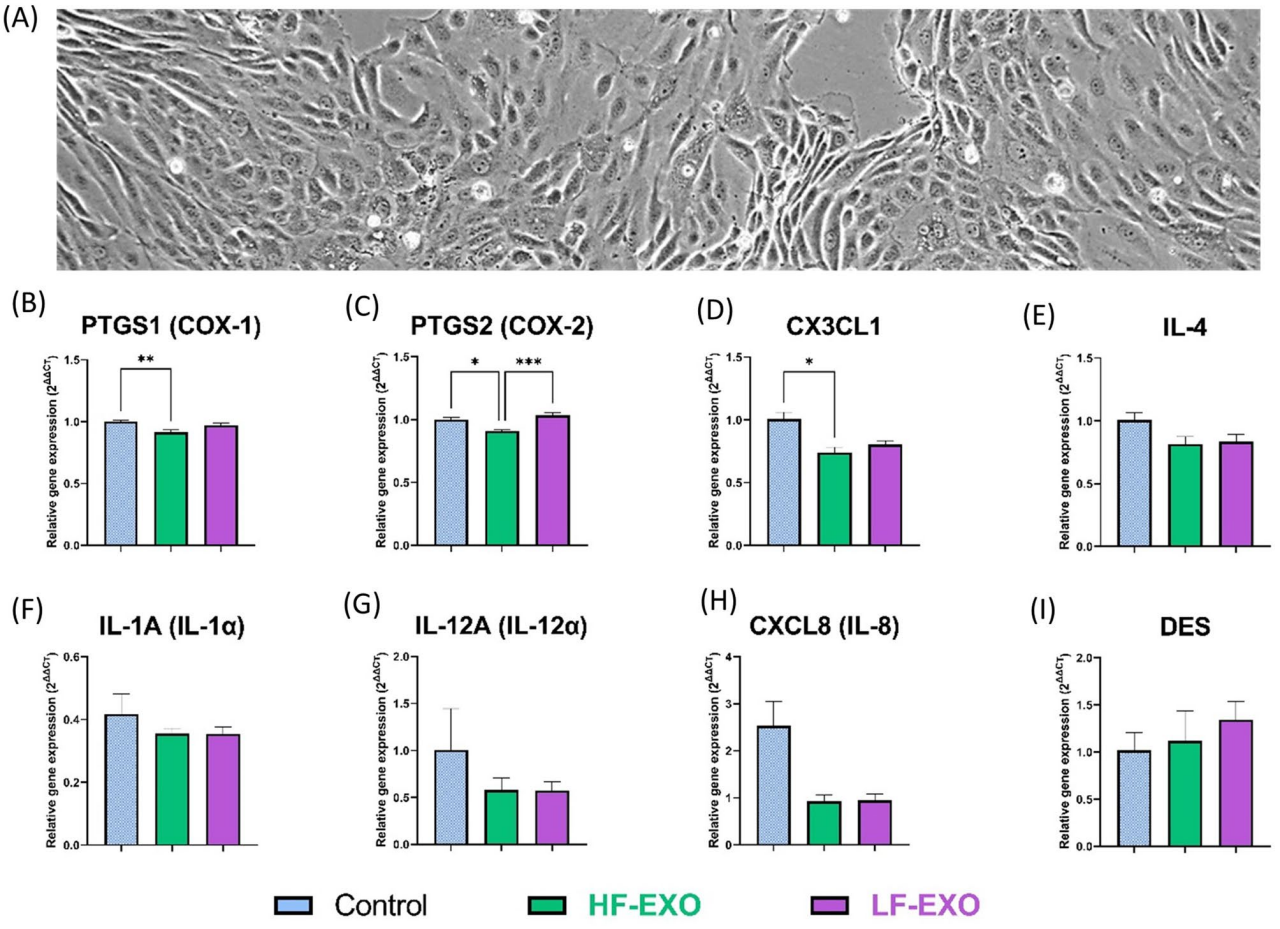
Co-incubation with sEV from dairy cows identified as high fertile (HF-EXO) and low fertile (LF-EXO) lead to differential gene expression in bovine endometrial epithelial (bEEL) cells. (**A**) bEEL cells depicts columnal or cuboidal morphology and showed contact inhibition after confluency (at 48-h, 10X). (**B,C**) Prostaglandin synthase enzyme gene expression, (**D,E**) anti-inflammatory mediator gene expression (**F,I**) pro-inflammatory mediator gene expression in bEEL cells. Values are presented as mean ± SEM. Brown-Frosythe and Welch ANOVA test along with Dunnett’s T3 multiple comparison was selected as post-hoc test to identify statistical significance differences between the groups. *P ≤ 0.05, **P ≤ 0.01, ***P ≤ 0.001.

To investigate the effect of blood plasma sEV on the endometrial epithelial tissue, bEEL cells (Fig. 3A) were exposed to HF-EXO and LF-EXO, in which the experimental set-up was similar to Section “LF-EXO shows higher total protein concentrations, however, did not show significant difference in total particle yield” bCSC cell experiments, and later, real-time quantitative PCR was performed to bEEL cells to identify inflammatory mediator gene expression levels (Fig. 3B–I).

Expression patterns of prostaglandin synthases PTGS1 and PTGS2 in bEEL cells were similar to bCSC cells (Fig. 3B,C). HF-EXO treatment has significantly downregulated both PTGS1 and PTGS2 levels against the untreated control. However, the rest of the pro-inflammatory cytokines did not show any profound downregulation of HF-EXO treated bEEL cells to the control (Fig. 3F–I). Interestingly, PTGS2 was the only gene significantly upregulated in bEEL after LF-EXO exposure (compared to the HF-EXO exposure) (Fig. 3C), which follows the trend of bCSC cells (Fig. 2C). Anti-inflammatory cytokine gene expression in bEEL cells exposed to either LF-EXO or HF-EXO were not profoundly changed against the control, however, CX3CL1 gene expression of bEEL cells exposed to HF-EXO was downregulated in contrast to the untreated control, suggesting a pro-inflammatory action rather anti-inflammatory function (Fig. 3D–E).

However, DES didn’t show any significant fold-change of gene expression between the groups (Fig. 3I).

Results for RNA quality analysis using Nanodrop are in Supplementary File 4. The C_T_ value calculations and summery of relative fold changes for the total set of genes (including genes illustrated in the main text) of the bovine array (Cat. no. 330171 CLAB39919) are in the Supplementary File 1.

### Gene enrichment analysis for differentially expressed genes

**Figure 4:**
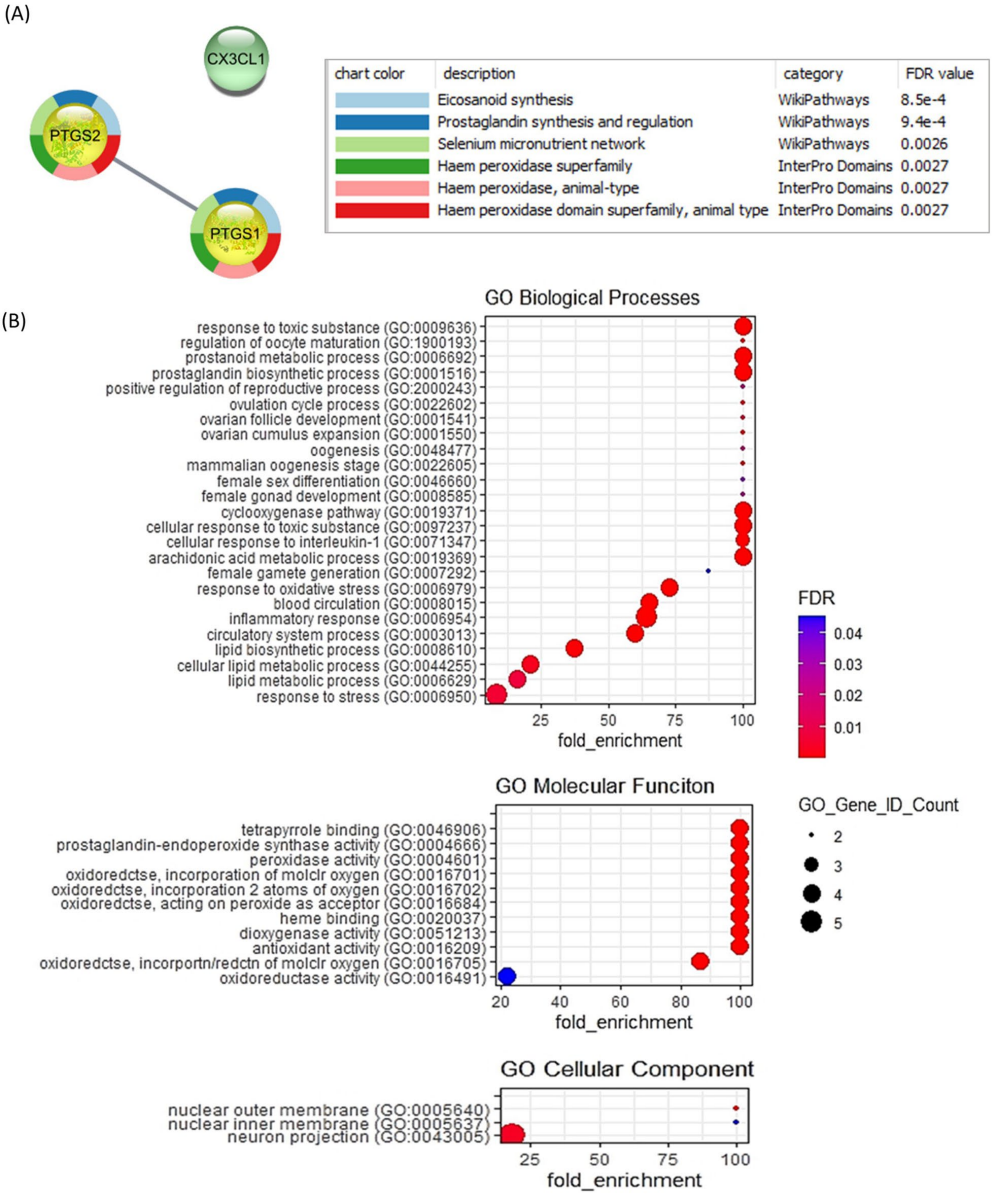
Gene enrichment analysis for 3 significantly downregulated genes (PTGS1, PTGS2 and CX3CL1) in bovine endometrial cells after incubation with HF-EXO compared to the control (**A**) STRING protein n-protein interaction (PPI) analysis (**B**) Gene ontology (GO) enrichment for biological processes, molecular function and cellular component revealed highest enriched GO terms relate to reproduction and inflammation. Fisher’s exact test was used for GO annotations. The corrected P-value was calculated to false discovery rate (FDR), FDR < 0.0001.

Collectively, PTGS1, PTGS2 and CX3CL1 displayed a significant downregulation in both bCSC and bEEL cells incubated with HF-EXO compared to the untreated control group. Therefore, these 3 genes were selected to identify enriched cellular mechanisms. STRING protein–protein interaction (PPI) analysis shows CX3CL1 acts independently to the connected genes PTGS1 and PTGS2. Further, WikiPathways and InterPro enrichment analysis conducted through Cytoscape (v3.9.0) confirmed involvement of PTGS1 and PTGS2 in eicosanoid, prostaglandin synthesis, and relationship to haem peroxidase protein (Fig. 4A). The gene ontology (GO) conducted demonstrated above three genes relates most to stress, inflammation and cytokine activity. PantherDB enrichment identified duplicate gene IDs for PTGS1 and PTGS2 (different orthologs of the same gene), thus the results represent 5 gene IDs (CX3CL1- PTN000387960, PTGS1–PTN000224626, PTN007546927, PTGS2–PTN002628507, PTN000224656) (Fig. 4B).

Therefore, we can elucidate that the downregulation of PTGS1, PTGS2 and CX3CL1 may favour in the bovine endometrium positively to indicates as the high fertility compared to cows with average fertility.

## Discussion

Quantification of inflammatory related gene expression in bovine endometrial cells determined incubation of circulating sEV derived from high-fertile and low-fertile treatment groups influenced differed expression levels of inflammation related genes. Compared to the untreated control, high fertile animals, and their blood plasma derived sEV demonstrated significant downregulation of prostaglandin synthases (PTGS1 and PTGS2) in both bCSC and bEEL cell types. Additionally, pro-inflammatory cytokines (IL-1α and DES) in bovine stromal cells demonstrated the similar trend. However, only a few genes show a distinct change in expression levels between HF-EXO and LF-EXO treatment groups, such as pro-inflammatory PTGS2, IL-12α and IL-8 in bCSC cells. Therefore, our results suggest that the blood plasma sEV may have multiple roles in the fertility state of the animals and recognition of circulating sEV as a biomarker candidate of fertility. Even subtle changes in pro-inflammatory cytokines levels in the endometrial cells which facilitate through systemic circulation of sEV may reflect as a switch of high or low fertile state.

Reproduction failure is strongly linked to anatomical obstructions in the endometrium which often manifest as endometrial inflammation^51^. Aberrant inflammation elevates the level of inflammatory mediator eicosanoid production in the endometrium^52^. Our results indicate a strong and significant low levels of prostaglandin synthases (PTGS1 and PTGS2) in high fertile animal endometrial stroma and epithelia, which favours receptivity along with pregnancy. Both PTGS1 and PTGS2 converts arachidonic acid (AA) to eicosanoids, prostaglandin E_2_ (PGE_2_) and prostaglandin-F2-alpha (PGF_2α_). Prostaglandin (PG) synthesis is a vital cellular activity during the ovulatory cascade while PGE_2_ considered as the key ovulatory PG regulator^53^ by enhancing the binding capacity of oocytes^54^. Nonetheless, PGE_2_ acts as an immunosuppressor during endometrial inflammation and infection^55^ and PGF_2α_ enhances embryo implantation and synthesize as an inflammatory response^56^. In our study, expression levels of PTGS1 and PTGS2 were similar in both endometrial cell lines, bCSC and bEEL, which is consistent with its function as a housekeeping agent. In a separate study on proteomic contents of sEV, absence of toll-like receptor 4 (TLR4) was observed in plasma sEV derived from dairy cows with high-risk to metabolic diseases in contrast to unique presence of TLR4 in plasma sEV derived from dairy cows with low-risk to metabolic diseases^17^. TLR4 is a critical protein involves in immune function which recognizes pathogens such as lipopolysaccharides from gram negative bacteria^57,58^. Interestingly, it has been reported that PGE_2_ reduces TLR4 expression^59^. Thus, a relationship with PTGS2 upregulation in both bCSC and bEEL after incubation of LF-EXO (Figs. 2C and 3C) and the low fertile origin of sEV may elucidate through PGE_2_ stimulation and immune suppression by inhibition of TLR4, in contrast to HF-EXO. On the other hand, downregulation of prostaglandin synthases in bCSC and bEEL cells exposed to HF-EXO may indicates a minimal inflammatory stimulus in the endometrium which predominantly expressed as the health states, specifically in the fertility of the animals.

Elevated cytokine expression mimics a pro-inflammatory state in the endometrium. This chronic condition results in anatomical abnormalities to the endometrial tissues including the epithelia and the stroma. Chronic inflammation can further develop into reproductive disorders such as endometritis, mastitis which negatively affect fertility and success of pregnancy. Among cytokines, interleukins play a critical reproductive functional role in the ovary and endometrium which involves in various pathophysiological processes. Interleukin-1-alpha (IL1α) regulates PG production and cyclooxygenase activity, particularly by stimulating PG production in endometrial stromal cells^60^. IL-8 along with IL1α shows elevated expression in bovine cystic ovarian disease which later causes infertility^61^. Nonetheless, IL-8 is a reproductive functional cytokine which is a mediator of follicular development, ovulation and corpus luteum function^62^. Our results show sEV derived from high fertile dairy cow downregulates IL1α compared to the control, IL-8 and IL-12α compared to the LF_EXO treatment in bovine endometrial stromal cells. It aligns with the inverse argument of relationship between low fertility and inflammation and similarly, indicates the suppression of a pro-inflammatory state in the high fertile animals which favours a healthy and a favourable endometrial stroma.

Desmin is an intermediate filament that plays a functional role in cellular remodelling and in mitochondrial distribution in striated muscles^63^. Desmin levels are elevated in human endometrial cells in early pregnancy and placentation to facilitate uterine remodelling, during which striated muscles transform the uterine tissues to a stiff and collagen filled extracellular matrix tissue^64^. In this study, differential expression of Desmin indicates a possible role of sEV from HF cattle in reducing inflammation in bCSC, which may in turn affect cellular integrity related to implantation and pregnancy. As Desmin levels may fluctuate during the oestrous cycle in cattle, this observation warrants further investigation of sEV cargo in relation to potential upstream effectors of Desmin contained therein.

In contrast, IL-4 is considered as anti-inflammatory cytokines which act antagonistically on pro-inflammatory cytokines^65^. The gene expression levels of IL-4 didn’t show any significant changes in both bCSC and bEEL cells exposed to sEV regardless of fertility states and even when compared to the control. These results are interesting because, simultaneous downregulation of pro-inflammatory mediator genes (PTGS2, IL-12α and IL-8) in HF-EXO compared to LF-EXO exposed endometrial stromal cells. Fractalkine (CX3CL1) considered as an anti-inflammatory^65^ and pro-inflammatory^66,67^ depending on the action pathway. Expression levels of CX3CL1 has downregulated in both bCSC and bEEL after incubation with HF-EXO compared to the control, in which we can elucidate a pro-inflammatory action of CX3CL1 on bovine endometrial cells. This suggests that sEV molecular cargo unrelated to fertility status may affect expression of CX3CL1 in the bovine endometrium. However, further studies will be required to understand the role of sEV on downregulating anti-inflammatory cytokines. Therefore, careful selection of inflammatory genes as biomarkers may assist in establishing a non-invasive methodology to identify fertility status.

Systemic blood flow is a critical supply channel of cellular signalling mediators specifically through sEV, which activates phenotypic changes throughout the entire organ system of an organism^68,69^. Plasma EV cargo facilitates critical functions specifically during reproduction and recent studies have identified EV miRNAs as a potential biomarker to identify super-stimulatory response during artificial insemination (AI) of cattle^70^. Nonetheless, proteomic content of circulating plasma exosomes have been identified to distinguish dairy cows with or without uterine infection^71^.Thus, we believe the selection of plasma sEV reflects an excellent cross-section of the physiology of the dairy cows. However, studies have analysed levels of inflammatory cytokine expression using EVs from other sources as well. Similar to our results of plasma HF-EXO on endometrial cells, milk derived exosomes have suppressed pro-inflammatory cytokines including PTGS2 on a macrophage cell line^72^. Further, umbilical cord-derived mesenchymal stem cells and its EVs have shown therapeutic value by decreasing pro-inflammatory cytokines to alleviate subclinical mastitis in dairy cow^73^.

The divergently fertile dairy cows selected in this study has previously been generated by mating dairy cows having polarized fertility traits i.e. a HF cow vs a HF bull vice versa^34,74^. The focus of our study was to utilize sEV from blood plasma collected during normal rearing conditions, thus blood from the animal groups selected to isolate plasma sEV in his study were collected before the pregnancy. However, it would be worthwhile to explore the inflammatory cytokine expression during the pregnancy, as there have been reports on changes to the exosome content during the pregnancy^31,32^.

Our study only focussed on the effects of plasma sEV from divergent fertility origin on endometrial cells, in which to explore the link to the reproduction system. However, it will be informative if the studies can expand to explore relationships of HF-EXO and LF-EXO on gut and nervous systems, as fertility affects nutrition, neurological and lifestyle factors as well^75^.

These findings highlight the unique properties of bCSC and bEEL cells given the origin of the cell types as well. The two endometrial cell types exhibit specific morphological and functional properties, such as different types of inflammatory responses^76,77^. Bovine endometrial stromal cells have been reported to produce more PGE_2_ and less PGF_2α_ when compared to bovine endometrial epithelial cells^76^. However, critically endometrial cell signalling pathways act collectively through the stroma to the epithelium during performing vital reproductive functions^78^. As our experiments confined to separate in vitro cell culture experiments of bEEL and bCSC cells, we propose performing a co-culture experiment for both bCSC and bEEL cells. That will enable understanding of the pro-inflammatory and anti-inflammatory cascade in endometrial epithelia and stroma collectively and will address the absence of mutual cell–cell communication due to separate in vitro cell culture experiments.

Our results demonstrate a switch-off state of inflammatory PG mediator gene expressions (PTGS1 and PTGS2) evident in high fertile animal using bovine endometrial in vitro cell culture model yet compared to the untreated controls. Therefore, the limited production of PGE_2_ and PGF_2α_ may facilitate favourable conditions for the receptivity of the endometrium of the high fertile animals. Suggested mechanism is attached in the Supplementary Fig. 3.2. Further, pro-inflammatory (IL-1A and DES) gene expression in endometrial stromal cells shows similar trend as well. However, LF-EXO exposure in endometrial stroma significantly upregulated few genes (PTGS2, IL-12α and IL-8). Collectively, our results explores interesting insights on the dynamics of inflammatory cytokine expression in the endometrium, in which both the bovine endometrial epithelial and stromal cells initiate pro-inflammatory cytokine expression, however only subtle changes were observed between HF-EXO and LF-EXO groups. Therefore, even the subtle changes on the inflammatory cascade in the endometrium may affect the fertility state. Therefore, sEV from high fertile animals acts in a unique direction to de-activate pro-inflammatory cytokines in endometrial stroma and epithelial to favour their high fertility state. In future, a thorough investigation into the functionality of sEV cargo specifically, miRNA and protein interactions could lead to a greater understanding on exosomal epigenetics and proteomics. A holistic view gained by the application of multiomics analysis will better elucidate the underlying mechanisms that drive divergent fertility states.

## Supplementary Information

Supplementary Tables.

Supplementary Tables.

Supplementary Figures.

Supplementary Tables.

## Data Availability

All the data are available online in Replication data to Prostaglandin synthases and pro-inflammatory cytokine gene expression dynamics in bovine endometrial cells exposed to cow blood plasma small extracellular vesicles (sEV) reflect the fertility breeding value—Harvard Dataverse^79^.
