# Supplementary material for: Dynamics of inflammatory cytokine expression in bovine endometrial cells exposed to cow blood plasma small extracellular vesicles (sEV) may reflect high fertility
Source: Sci Rep. 2023 Apr 3;13:5425. doi: 10.1038/s41598-023-32045-1 (PMC10070242; doi:10.1038/s41598-023-32045-1)

Supplementary file 3

Prostaglandin synthases and pro-inflammatory cytokine gene expression dynamics in bovine endometrial cells exposed to cow blood plasma small extracellular vesicles (sEV) reflect the fertility breeding value.

Pevindu Abeysinghe, Natalie Turner, Eman Mosaad, Jayden Logan and Murray D. Mitchell

Supplementary figure 3.1 Western Blot results

- BSA

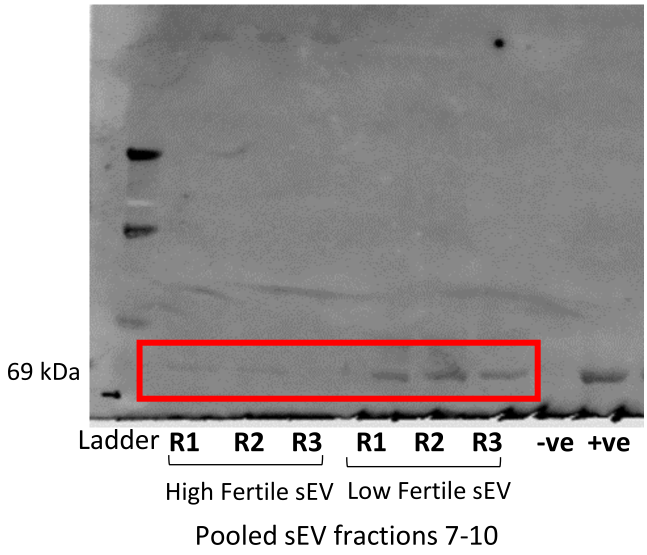

- Flotillin -1

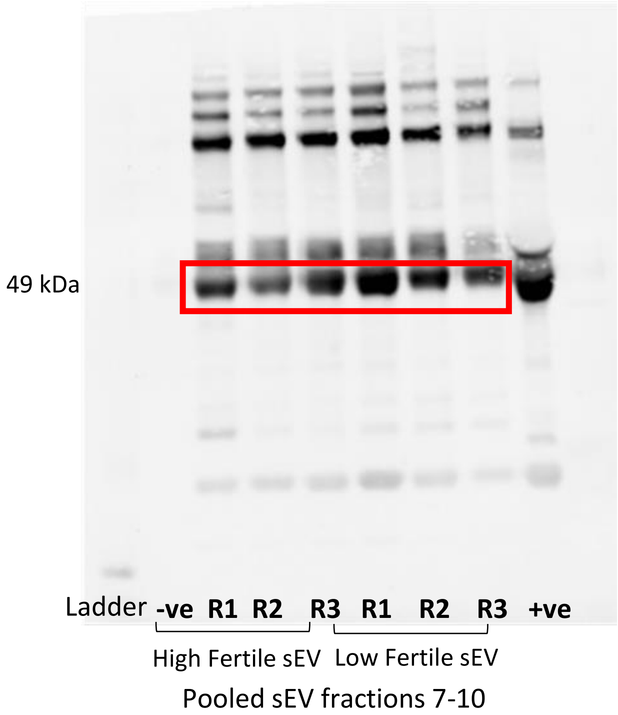

- CD81

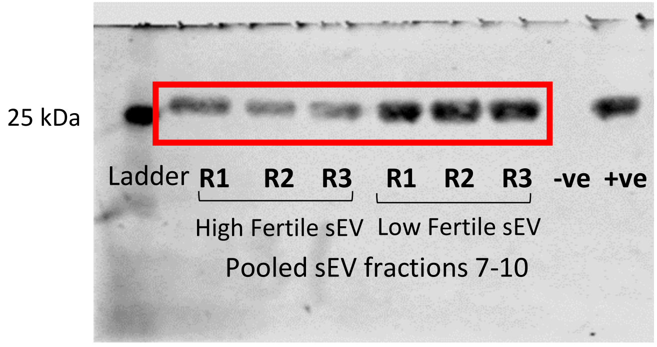

*R1, R2 and R3 are technical replicates.

Supplementary figure 3.2

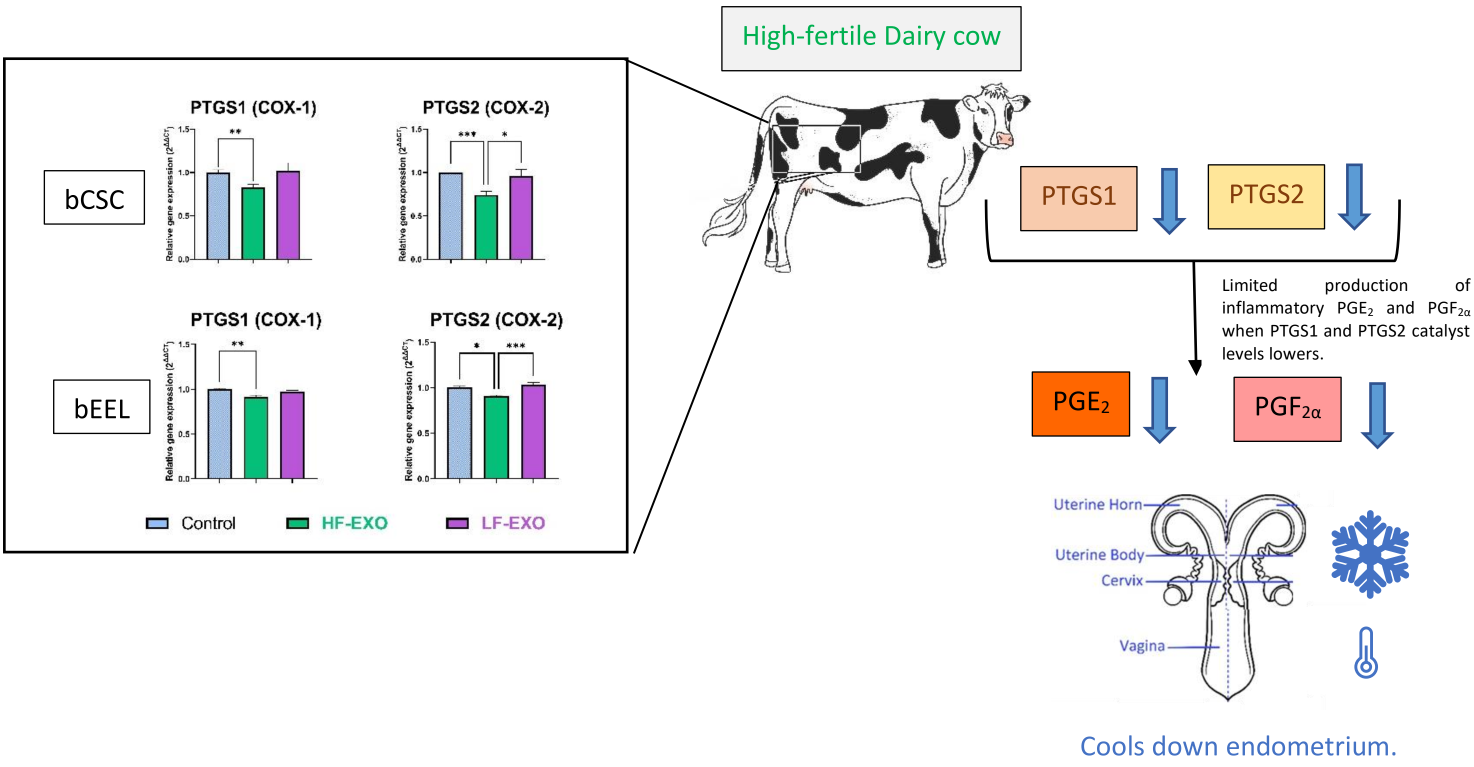

Supplement: Supplementary file 3 — Supplementary Figures. [file 41598_2023_32045_MOESM3_ESM.pdf]
